# Therapy of Chronic Myeloid Leukemia: Twilight of the Imatinib Era?

**DOI:** 10.1155/2014/596483

**Published:** 2014-01-30

**Authors:** Ewelina Trela, Sylwester Glowacki, Janusz Błasiak

**Affiliations:** Department of Molecular Genetics, Faculty of Biology and Environmental Protection, University of Lodz, Pomorska 141/143, 90-236 Lodz, Poland

## Abstract

Chronic myeloid leukemia (CML) results from the clonal expansion of pluripotent hematopoietic stem cells containing the active *BCR/ABL* fusion gene produced by a reciprocal translocation of the *ABL1* gene to the *BCR* gene. The BCR/ABL protein displays a constitutive tyrosine kinase activity and confers on leukemic cells growth and proliferation advantage and resistance to apoptosis. Introduction of imatinib (IM) and other tyrosine kinase inhibitors (TKIs) has radically improved the outcome of patients with CML and some other diseases with *BCR/ABL* expression. However, a fraction of CML patients presents with resistance to this drug. Regardless of clinical profits of IM, there are several drawbacks associated with its use, including lack of eradication of the malignant clone and increasing relapse rate resulting from long-term therapy, resistance, and intolerance. Second and third generations of TKIs have been developed to break IM resistance. Clinical studies revealed that the introduction of second-generation TKIs has improved the overall survival of CML patients; however, some with specific mutations such as T315I remain resistant. Second-generation TKIs may completely replace imatinib in perspective CML therapy, and addition of third-generation inhibitors may overcome resistance induced by every form of point mutations.

## 1. Introduction

Chronic myeloid leukemia (CML) was a fatal disorder until the introduction of imatinib (IM, also known as STI571, Gleevec, Glivec), which revolutionized its therapy, changing CML into a chronic disease. This was the advent of the “imatinib era.” IM is a model drug of targeted cancer therapy, in which only cancer phenotype, associated with BCR/ABL enhanced tyrosine kinase activity, was affected and normal features were unchanged. This gave new hope for avoidance of unwanted side effects, which are usually associated with the use of chemotherapeutic drugs. However, some patients do not tolerate IM and some display resistance to it, resulting in increasing doses of the drug associated with its increased toxicity. To fight IM resistance several other tyrosine kinase inhibitors have been worked out, but soon it became clear that they have several advantages over IM and may completely replace it in CML therapy.

## 2. Chronic Myeloid Leukemia: pathogenesis and Treatment 

CML is characterized by the expansion of bone marrow CML stem cell progeny. In 1960 Rudkin et al. detected a consistent chromosomal abnormality characteristic of CML, which later was named as the Philadelphia (Ph) chromosome [1, 2].

**Figure 1:**
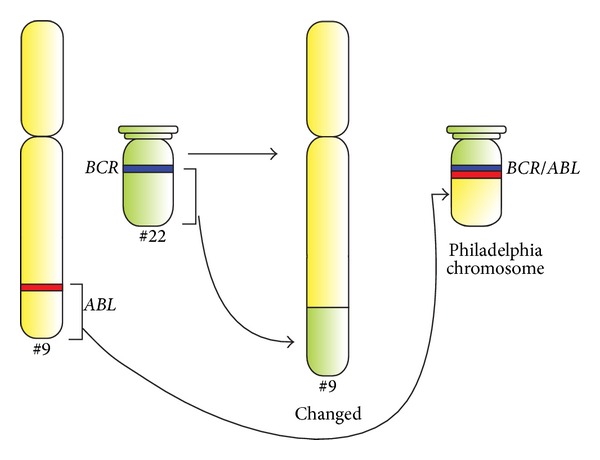
Schematic diagram of the translocation that forms the Philadelphia chromosome and schematic representation of the *BCR* and *ABL* genes. The *ABL* and *BCR* genes reside on the long arms of the chromosomes 9 and 22, respectively. The fusion *BCR/ABL* gene is formed within the derivative Philadelphia chromosome as a result of the (9; 22) translocation.

The Ph chromosome results from a reciprocal translocation, which involves the *ABL* protooncogene on the chromosome 9 and *BCR* (breakpoint cluster region) on chromosome 22, t(9; 22)(q34; q11) (Figure 1) [3]. This translocation creates the *BCR/ABL* fusion gene, which is believed to be the principal cause of CML and is considered as a hallmark of this disease [4]. Depending on the breakpoint in the *BCR* gene, three main types of the fusion proteins are formed: p210^*BCR/ABL*^ (M-bcr breakpoint), which is the most common in CML, p230^*BCR/ABL*^ (*μ*-bcr breakpoint), and p190^*BCR/ABL*^ (M-bcr breakpoint) (Figure 1). The latter is observed in two-thirds of acute lymphoblastic leukemias (ALLs) and in very rare cases of CML and acute myeloid leukemia (AML) patients. The native c-ABL kinase is located mainly in the nucleus, whereas the BCR/ABL fusion protein is found in the cytoplasm [5]. CML is unusual among human cancers because the expression of chimeric active BCR/ABL protein is thought to be the causative molecular event of CML [4]. The normal tyrosine kinase activity of the ABL protein is tightly regulated, but it may change into constitutive activity by the juxtaposition of the BCR sequence in the BCR/ABL protein (for more details see [6]). In this way, BCR/ABL is able to transduce signals in various pathways in an autonomous fashion through the phosphorylation of a number of substrates [7]. BCR/ABL triggers multiple downstream pathways leading to enhanced cell proliferation and transformation, reduced growth factor dependence, resistance to apoptosis, perturbed adhesion to bone marrow and stroma, and genetic instability [2]. This results in the expansion of the leukemic cell population, initially characterized by overproduction of mature myeloid cells with normal morphology [8]. Many of BCR/ABL substrates and binding partners were identified and current efforts are directed at linking these pathways to specific defects, typical for CML [4, 9, 10].

CML is diagnosed in 1 to 2 cases per 100,000 per year, usually in its initial phase, called chronic phase (CP), when functionally normal mature blood cells are produced [2, 11]. Patients with CML may present night sweats, fatigue, abdominal fullness, gout, leukocytosis, and splenomegaly [2, 12]. The median age at diagnosis of CML is 50 to 55 years [13, 14]. After three to five years, the disease advances an accelerated phase (AP) if not treated. This stage is characterized by an increase in disease burden and the occurrence of progenitor/precursor cells rather than terminally differentiated cells. Finally, the last CML phase is blast crisis (BC), characterized by rapid expansion of myeloid differentiation-arrested blast cells [11, 15]. In BC, the disease resembles AML (two-thirds of cases) or ALL (one-third) [2]. CML is one of a few malignant diseases triggered by the BCR/ABL protein with its constitutive tyrosine kinase activity. It soon became clear that the essential role of BCR/ABL tyrosine kinase activity responsible for cell transformation might be rational for targeting this function therapeutically. Before the introduction of the tyrosine kinase inhibitor imatinib mesylate, conventional treatment for CML patients involved spleen irradiation, hydroxycarbamide, and busulfan or interferon-alpha. Of these, only interferon-alpha induced cytogenetic responses (≤35% Ph-positive metaphases) in around 20% of patients and prolonged the duration of chronic phase and survival compared with conventional chemotherapy [2, 7, 16]. Subsequent efforts have focused on the design of compounds with chemical properties that compete with ATP, which is a cofactor for BCR/ABL.

**Figure 2:**
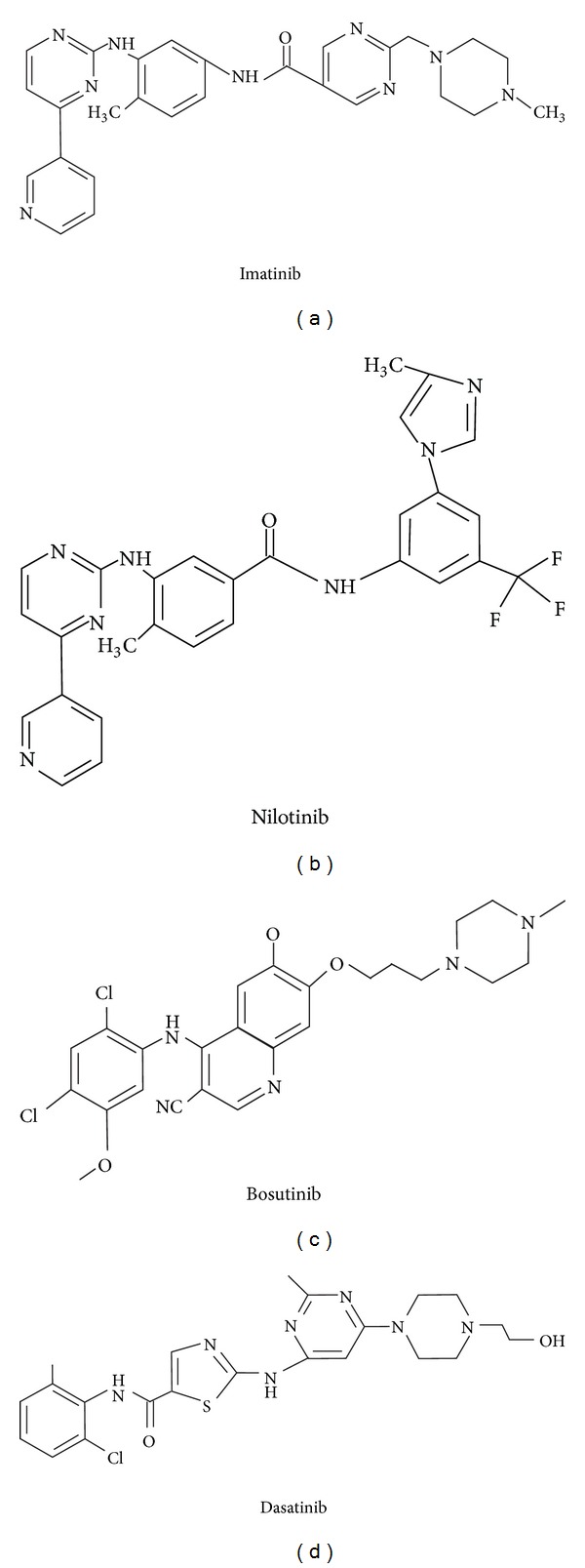
Molecular structures of imatinib, nilotinib, bosutinib, and dasatinib.

The most successful synthetic ATP-binding inhibitor that has been developed to date is IM (2-phenylamino-pyrimidine derivative, C_30_H_35_N_7_SO_4_, MW 589.7), formerly known as STI571 (signal transduction inhibitor number 571; Glivec, Novartis Pharma) (Figure 2) [16, 17]. IM was approved for CML treatment by the US Food and Drug Administration (FDA) in May 2001 and radically improved the outcome of patients due to its remarkable activity and mild toxicity [18]. The most striking feature of IM is its high degree of BCR/ABL specificity, while its effect on other tyrosine kinases is negligible [19, 20]. The vast majority of patients treated with IM have cytogenetic and even molecular responses (undetectable BCR/ABL transcripts by reverse transcriptase polymerase chain reaction) [2, 21]. Despite the improved survival observed with IM use, a proportion of patients has a primary resistance to IM or develops it in the course of therapy or cannot tolerate this drug [19].

## 3. Imatinib Resistance

Imatinib, at micromolar concentrations, inhibits the kinase activity of all proteins that contain ABL, ABL-related gene protein, or platelet-derived growth factor receptor (PDGFR), as well as the KIT receptor [22–24].

**Figure 3:**
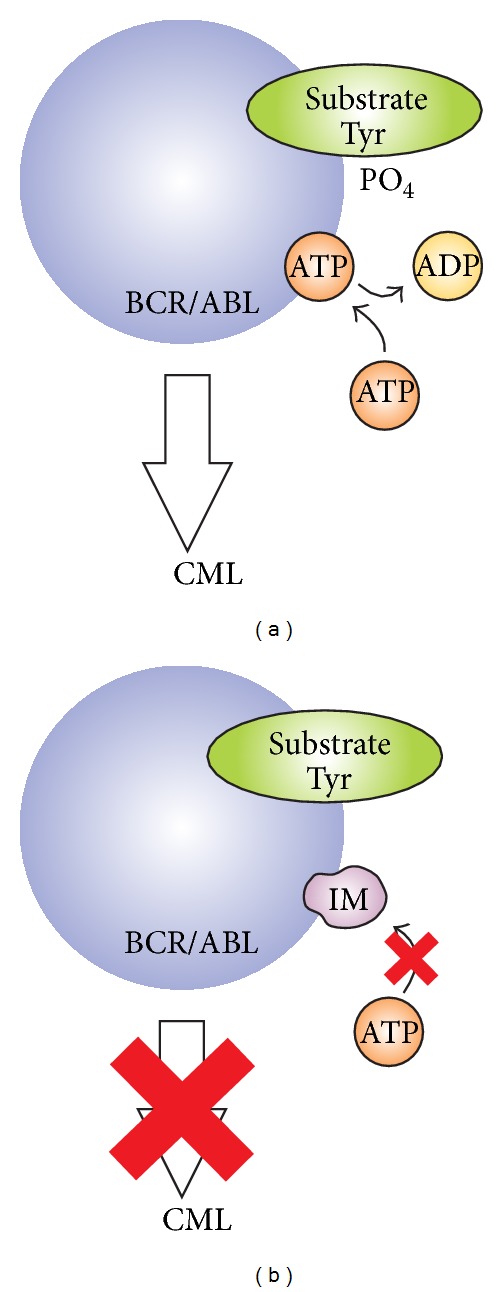
Schematic mechanism of IM action. (a) The constitutively active BCR/ABL tyrosine kinase functions by transferring phosphate from ATP to tyrosine residues on various substrates leading to excess proliferation of myeloid cells typical for CML. (b) IM blocks binding of ATP to the BCR/ABL kinase, causing inhibition of its activity.

IM binds to BCR/ABL ATP-binding site and stabilizes inactive non-ATP-binding form of BCR/ABL, thus preventing tyrosine autophosphorylation and, in turn, phosphorylation of its substrates (Figure 3) [16, 24]. IM inhibits cellular growth and induces apoptosis in CML, both *in vitro* and *in vivo* [22]. In the IRIS study (International Randomized Study of Interferon versus IM) of first-line treatment with IM or interferon and cytarabine in patients with newly diagnosed chronic phase, CML patients treated with IM had an 8-year overall survival rate of 85% and lack of progression to advanced disease was 92% [19, 25]. However, although IM induces cytogenetic responses in majority of patients, the emergence of resistance to IM was recognized as a major problem in the treatment of Ph-positive leukemias [24, 26]. A minority of CML patients in CP and a substantial proportion in AP and BC are either initially refractory to IM or lose IM sensitivity over time and experience relapse, or cannot tolerate side effects of the drug [20, 27]. Therapeutic resistance to IM is seen in approximately 10–15% of patients and can be classified as primary or secondary, according to the time of onset [28]. Primary (intrinsic) resistance is a lack of efficacy from the onset of treatment with IM and secondary (acquired) resistance is defined as an initial response followed by a loss of efficacy with the time of exposure to IM [29, 30]. Clinically, it would be advantageous to identify patients prior to the resistance onset, since they may benefit from more aggressive therapy [31, 32].

Some mechanisms leading to IM resistance have been characterized. Amplification of the *BCR/ABL* gene and overexpression of the BCR/ABL protein were shown to underlay IM resistance both *in vitro* and *in vivo*. Moreover, mutations in the BCR/ABL domain can confer IM resistance, either by altering IM binding characteristics or through indirect modulation of kinase function. These mechanisms are often associated with secondary (acquired) resistance [28]. In this sense, kinase domain mutations are the most commonly identified mechanism associated with relapse and the substitution of threonine with isoleucine at residue 315 (T315I) was the most frequently observed mutation in IM-resistant patient [33]. However, it was reported that none of 12 CML patients screened for mutation in the BCR/ABL kinase domain had the T315I mutation and only one patient had a point mutation [34]. It is consistent with an other study which showed that mutations conferring resistance to IM, including T315I, were found infrequently in them [20]. Another study showed that P-loop mutations are not associated with poor outcome and suggested that the prognosis was dependent on several other factors [35]. It becomes evident that the presence of mutations does not explain all cases of IM resistance and the emerging problem is the primary resistance associated with BCR/ABL-independent mechanisms [16, 23]. Factors contributing to primary resistance are poorly known and investigation of processes underlying it has begun [28]. It was stated that although IM is successfully used in the treatment of CML, inherent mechanisms confer primary resistance in leukemic patients [36]. Moreover, even in cases of the most successful courses of therapy, IM treatment does not eradicate all leukemic cells. To prevent disease relapse, a continuous supply of the drug is needed [20]. Should IM therapy be halted, population of leukemic cells rebuilds and disease symptoms return. It was suggested that there is a reservoir of primitive, quiescent leukemic stem cells that are unresponsive to IM treatment and can give a beginning to a new population of leukemic cells in the absence of IM [37, 38].

There is an increasing body of evidence demonstrating that the malignant phenotype results from gaining of both genetic abnormalities and epigenetic modifications with time [39]. Epigenetics is characterized by the heritable changes in the patterns of gene expression that occur without a change in the primary DNA sequence [13]. In this context, epigenetic alterations, including DNA methylation, may have an impact on CML progression and resistance to IM. Indeed, DNA hypermethylation was previously seen in various types of leukemia [40]. It was reported that DNA methyltransferases (DNMTs) are overexpressed in leukemic cells in a leukemia type- and stage-specific manner and thus upregulated DNMTs may contribute to the pathogenesis of CML [41, 42]. This is consistent with the data presented by Jelinek et al. [40]. They found that the average number of methylated genes was 4.5 per patient in the CP, increasing to 6.2 in the AP and 6.4 in the BC. A higher number of methylated genes were also observed in patients resistant or intolerant to IM. Thus, DNA methylation is strongly associated with CML progression and resistance to IM. Other studies concentrated mainly on genes whose expression differs between responders and nonresponders to IM [26, 28, 36, 43]. Results of another study suggest that the transcriptional regulation of apoptotic and antiapoptotic genes, disease progression genes, oxidative stress genes, genes for DNA repair and genes whose products are known to interact with centrosomes is associated with IM resistance in CP CML patients [36]. Moreover, genes involved in cell adhesion, drug metabolism, protein tyrosine kinases, and phosphatases were found to be expressed differentially in patients sensitive and resistant to IM [44]. The expression of the *hOCT1* gene, a drug transporter gene, was also indicated to have influence on intracellular concentration of IM and, thus, patients may fail to achieve a cytogenetic response [45]. However, further work to explore the interaction of hOCT1 and other drug transporters as a cause of primary cytogenetic resistance to IM is needed [46]. Many other genes, including hypermethylation of an autophagy-related gene *ATG16L2* and downregulation of the *BIM* gene were associated with poorer prognosis in terms of molecular response to IM treatment [47–49]. These data suggest that primary resistance to IM is mediated through complex mechanisms, which are largely BCR/ABL independent. It was also suggested that the resistance to IM may be multifactorial [50]. Therefore, the idea of combined treatment with demethylating agents seems to be justified [13, 39, 41, 51].

It is known that a critical goal of IM activity is to eliminate BCR/ABL-expressing cells [52]. Mammals have two distinct apoptosis-signaling pathways. One is triggered by ligation of members of the TNF-R (tumor-necrosis factor receptor) family with an intracellular “death domain” and requires FADD (Fas-associated death domain protein) mediated activation of caspase-8. The other is activated by developmental signals or certain cytotoxic drugs. This pathway is regulated by the interplay of pro- and antiapoptotic members of the Bcl-2 protein family and involves mitochondrial release of apoptogenic molecules for caspase activation and cell destruction [53]. The Bcl-2 protein family contains three major subgroups. The first subgroup includes Bcl-2, Bcl-X_L_, and Mcl-1, which play a role as antiapoptotic proteins. The second subgroup includes Bax and Bak, and these proteins are essential for cell death [53, 54]. The third subgroup includes Bim, Bad, Bmf, Noxa, and Puma, which are known to act by neutralizing the antiapoptotic proteins [54]. IM was shown to induce apoptosis through Bim accumulation independently of cell cycle arrest [55]. It was reported that IM activates not only Bim, but also Bad and Bmf. In addition, the same study revealed that Bim and Bad account for most IM-induced apoptosis of BCR/ABL cells. In this context, Bim may play an essential role in IM-induced apoptosis and overexpression of antiapoptotic proteins of Bcl-2 family may contribute to IM resistance. Indeed, it was shown that IM-resistant cells demonstrate Bcl-2 overexpression, whereas primary CML cells expressed significantly lower amounts of Bim compared with normal bone marrow cells [53, 56]. Therefore, it would be justified to combine IM treatment with molecules which mimic BH3 proteins (the third subgroup of Bcl-2 protein family) and trigger apoptosis as an all-or-nothing process [40, 57]. It was reported that such combination can lead to an enhancement of IM-induced apoptosis [53, 56]. Moreover, it was shown that a combined treatment with nutlin-3 and IM activates p53 without inducing p21 and synergistically activates Bax-mediated mitochondrial pathway to induce apoptosis in BCR/ABL-expressing cells [58]. This finding is especially important because apoptosis was observed in primary leukemic cells from patients with CML BC and cells expressing the IM-resistant E255 K mutation, suggesting that patients with IM-resistance mutation may benefit from combined treatment. Different study reported that nearly complete elimination of phenotypically and functionally defined CML stem cells is possible through combination of IM with inhibitors of autophagy [59]. Autophagy is a degradative process in eukaryotic cells which results in the breakdown of damaged or not needed intracellular material within lysosomes under homeostatic conditions or in response to stress signals, allowing cells to adapt to environmental and/or developmental signals [59]. Taken together, these results suggest that combined treatment with IM and other molecules that enhance IM-induced apoptosis may be an option for CML patients who are IM resistant.

## 4. Second Generation of Tyrosine Kinase Inhibitors

New BCR/ABL inhibitors, such as nilotinib, dasatinib, or bosutinib, were developed to overcome emerging problem with IM resistance [60]. Like IM, second-generation TKIs are orally administered and bind to the ATP-binding site of BCR/ABL [61].

Nilotinib (N-(3-(3-(1H-imidazolyl)propoxy)phenyl)-4-methyl-3-((4-(3-pyridinyl)-2-pyrimidinyl)amino) benzamide; Tasigna; Novartis Pharmaceuticals) (Figure 2) was developed through chemical modification of IM and has a similar structure to a 30-fold higher potency against BCR/ABL *in vitro* [62]. It has been approved for the treatment of CML patients in CP or AP and for those who are resistant to or intolerant of IM or other prior therapies [32, 63, 64]. Nilotinib has nearly identical binding site within ABL as IM but requires fewer hydrogen bonds (4 versus 6), enabling binding to numerous IM-resistant BCR/ABL mutants [61]. However there are still some BCR/ABL mutations that confer resistance to nilotinib, including F317L, V299L, and T315A [65]. Nilotinib has a similar half-life (approximately 17 hours) to IM [61]. The improved binding of nilotinib results in a greater potency and selectivity over the Arg (ABL2), Kit, and PDGFR kinases but has no activity against Src family kinases [62, 66]. However, such activity is displayed by another BCR/ABL inhibitor approved for the CML treatment, dasatinib (N-(2-chloro-6-methylphenyl)-2-(6-(4-(2-hydroxyethyl) piperazin-1-yl)-2-methylpyrimidin-4-ylamino) thiazole-5-carboxamide); Bristol-Myers Squibb) (Figure 2) [67]. It is an orally available ABL kinase inhibitor that differs from IM as it can bind to both the active and inactive conformations of the ABL kinase domain [68]. Dasatinib is also active against Kit, PDGFR, and ephrin receptor [64]. In addition, dasatinib binds to other tyrosine and serine/threonine kinases, such as the TEC family kinases, the mitogen-activated protein kinases, and the receptor tyrosine kinase, discoidin domain receptor 1 [66]. Since dasatinib has less stringent binding requirements than IM, it is active against many IM-insensitive kinase domain mutations of BCR/ABL, with some exceptions including T315I and F317V/L [69]. Dasatinib is effective in patients previously treated with IM and has a manageable safety profile in all phases of CML. It is structurally unrelated to IM and has a shorter half-life than IM (approximately 3.6 hours) and no highly active metabolites [61, 64]. In addition, dasatinib is not a substrate for the P-glycoprotein efflux pump and therefore may be able to achieve higher intracellular concentrations than IM [70].

Bosutinib (4-anilino-3-quinolinecarbonitrile; Wyeth), an agent in preclinical trials, is a dual inhibitor of Src and ABL kinases (Figure 2) [21, 64]. Bosutinib is able to bind to both inactive and intermediate conformations of BCR/ABL [64, 66]. Bosutinib inhibits a broader range of kinase targets than IM or nilotinib, including Src family kinases, but had no significant activity against Kit or PDGFR [61, 66]. It also demonstrated activity against a number of mutations, but not T315I and V299L [71]. Bosutinib has a half-life of 22 to 27 hours and was shown to be effective in patients previously treated with dasatinib or nilotinib [61, 64, 66, 72].

There are several clinical trials on the efficacy and safety of second generation of TKIs in the treatment of CML patients, including “Evaluating Nilotinib Efficacy and Safety in Clinical Trials-Newly Diagnosed Patients” (ENESTnd), “Dasatinib versus Imatinib Study in Treatment-Naive CML Patients” (DASISION), and “Bosutinib versus Imatinib in Patients with Chronic Phase Chronic Myeloid Leukemia” (BELA) [21]. Direct comparison of the results from those trials is difficult because ENESTnd, DASISION, and BELA investigations had different study design, primary endpoints, and definitions. However, the data obtained from these trials suggest that higher rates of molecular response are achieved with nilotinib, dasatinib, and bosutinib when compared to IM [21]. Despite the higher rates of molecular response, T315I mutation is emerging as a common mechanism of failure of second-line TKIs [73]. It was reported that combined treatment with nilotinib or dasatinib with SGX393 (inhibitor of native and T315I-mutant BCR/ABL) might be useful for reduction of BCR/ABL mutants in Ph-chromosome-positive leukemia [73]. In addition, it was shown that combined treatment with dasatinib and vorinostat (suberoylanilide hydroxamic acid (SAHA) histone deacetylase inhibitor) led to depletion of wild-type and mutant forms of BCR/ABL-expressing cells [74]. Furthermore, it was indicated that combined ABL inhibitor therapy (dasatinib and IM) is a feasible treatment strategy for patients with CML [72]. Nevertheless, further studies and clinical trial of these drug combinations should be pursued.

## 5. Third Generation of Tyrosine Kinase Inhibitors and Other Agents in Postimatinib Era

Emerging mutations such as T315I, which renders both IM and second-generation TKIs, inspired research on third generation of TKIs that could act in patients with mutations that confer resistance to IM. To date one new agent was approved for clinical use: ponatinib. It was demonstrated that ponatinib is effective on CML cells harboring different mutations in BCR/ABL, including highly multiresistant T315I [75]. Ponatinib inhibits also other kinases beyond BCR/ABL such as PDGFR, FGFR, KIT, RET, and FLT3 [76, 77]. It was proven as potent drug in therapy of patients with advanced phase of CML [78].

Rebastinib (also known as DCC-2036) is another ABL kinase inhibitor that can overcome resistance to T315I mutation [79]. It was under first phase clinical trials for use in CML therapy where it demonstrated ability to induce in some patients cytological responses in chronic phase, as well as in accelerated phase [80].

Some other agents that do not function as kinase inhibitors are currently tested in BCR/ABL-dependent leukemias therapy. For example, aurora kinase inhibitor VX-680 showed activity against BCR/ABL positive leukemic cells. In addition it demonstrated a synergistic interaction with dasatinib, both drugs exhibiting higher cytotoxicity together than each drug singly [81].

It is important to note that although some second-generation TKIs can attack earlier progenitor cells when compared to IM, it is not known whether they can completely eradicate leukemic stem cells. Thus, therapies with second-generation TKIs cannot cure CML. However, it was proposed that autophagy inhibitors may render dormant leukemic cells susceptible to TKI action. One such research is currently run as CHOICES trial, in which hydroxychloroquine combined with IM is evaluated [82].

## 6. Conclusions

IM revolutionized CML treatment. Despite satisfactory outcome, it faced the emerging problem of resistance. Second-generation TKIs opened an alternative to IM treatment option for CML and other diseases with TKIs expression. Does it mean this is the end of IM era? On the one hand, novel second-generation TKIs are more potent than IM but remain insensitive to T315I mutation. However, if a patient has a non-T315I mutation, a second-generation TKI is warranted. On the other hand, great proportion of patients develops resistance to IM, but combinatory treatment seems promising to solve the problem of IM resistance. This can be projected to second-generation TKIs and the use of histone deacetylase inhibitors and aurora kinase inhibitors seems to be promising. Nilotinib is generally well tolerated and is not associated with unwanted side effects, typical for TKIs. However, it should be also taken into account that second-generation TKIs are more expensive than IM. In case of T315I harboring patients, recent development of third-generation TKIs specifically designed to target this mutation seems promising and may radically improve the situation of resistant patients. Certainly, this is not the end of the imatinib era, but a twilight might have begun.
